# Gut-Derived Metabolite, Trimethylamine-N-oxide (TMAO) in Cardio-Metabolic Diseases: Detection, Mechanism, and Potential Therapeutics

**DOI:** 10.3390/ph16040504

**Published:** 2023-03-28

**Authors:** Meyammai Shanmugham, Sophie Bellanger, Chen Huei Leo

**Affiliations:** 1 Science, Math & Technology, Singapore University of Technology & Design, 8 Somapah Road, Singapore 487372, Singapore; 2 A*STAR Skin Research Labs, Agency for Science, Technology and Research, Singapore 138648, Singapore

**Keywords:** TMAO, endothelial dysfunction, cardio-metabolic diseases, inflammation, oxidative stress

## Abstract

Trimethylamine N-oxide (TMAO) is a biologically active gut microbiome-derived dietary metabolite. Recent studies have shown that high circulating plasma TMAO levels are closely associated with diseases such as atherosclerosis and hypertension, and metabolic disorders such as diabetes and hyperlipidemia, contributing to endothelial dysfunction. There is a growing interest to understand the mechanisms underlying TMAO-induced endothelial dysfunction in cardio-metabolic diseases. Endothelial dysfunction mediated by TMAO is mainly driven by inflammation and oxidative stress, which includes: (1) activation of foam cells; (2) upregulation of cytokines and adhesion molecules; (3) increased production of reactive oxygen species (ROS); (4) platelet hyperreactivity; and (5) reduced vascular tone. In this review, we summarize the potential roles of TMAO in inducing endothelial dysfunction and the mechanisms leading to the pathogenesis and progression of associated disease conditions. We also discuss the potential therapeutic strategies for the treatment of TMAO-induced endothelial dysfunction in cardio-metabolic diseases.

## 1. Introduction

The endothelium is a monolayer of cells that lines the interior surface of the blood vessel and forms a partially permeable barrier between endothelial tissues and blood circulation. Blood vessels, comprising endothelial cells and vascular smooth muscle cells (VSMCs), serve essential secretory, synthetic, metabolic, and immunological roles [1]. Normal physiological conditions of the endothelium regulate vascular homeostasis by modulating vascular tone, platelet adhesion, inflammation, plasmatic coagulation, fibrinolysis, and VSMC proliferation. The generation and release of vasoactive factors by endothelial cells, such as endothelium-derived relaxing factors (EDRFs) and contracting factors (EDCFs), are vital for the maintenance of normal physiological conditions, and disturbances to these factors are known to increase the incidence of endothelial dysfunction [2,3,4]. Endothelial dysfunction, a pathophysiological condition wherein the endothelial homeostasis is disrupted, enhances the risk of thrombosis, inflammation, angiospasm, and intraplaque hemorrhage, resulting in atherothrombosis, infraction, and ischemia [1], and contributes to cardio-metabolic diseases, such as atherosclerosis, acute coronary syndromes, hypertension, reproductive disorders, and diabetes [5,6]. Multiple factors trigger endothelial dysfunction, which includes high blood pressure, cholesterol levels, genetics, and lifestyle practices such as smoking, physical inactivity, and diet. According to the Global Burden of Disease, Injuries, and Risk Factor study 2013, dietary risks are one of the most significant factors that contribute to cardio-metabolic diseases [7].

In recent years, trimethylamine N-oxide (TMAO) was found to be closely associated with cardio-metabolic diseases mediated through endothelial dysfunction. TMAO is a biologically active compound from a class of amine oxides, generated from dietary precursors highly enriched in red meat, fish, and egg yolk [8]. Studies have shown that plasma TMAO levels are elevated in individuals with type II diabetes [9], diastolic dysfunction [10], heart failure [10], atherosclerotic plaque deposition [11,12], and peripheral artery disease (PAD) [13]. Subsequent mechanistic studies revealed that TMAO treatment elevates inflammation and oxidative stress, which triggers cardio-metabolic diseases [14,15]. Given its well-established association with chronic inflammation and accelerated progression of cardio-metabolic diseases, TMAO has recently gained significant scientific interest as a potential circulating biomarker for predicting cardio-metabolic diseases and chronic kidney diseases (CKD) [16].

In this review, we discuss the currently available methods for TMAO detection and its known association with disease conditions. Furthermore, the molecular mechanisms of TMAO-induced endothelial dysfunction in experimental and clinical studies, as well as potential treatment strategies to prevent the progression of diseases triggered by TMAO, are also summarized.

## 2. TMAO Metabolism, Biosynthesis, and Excretion

**Figure 1:**
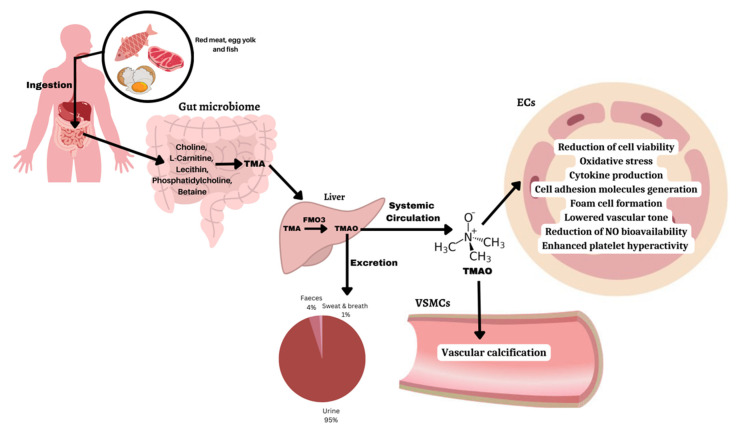
Biochemical pathways involved in the formation of TMAO. TMAO is synthesized from dietary precursors after the action of the gut microbiota and flavin-containing monooxygenases, mainly the FMO3 enzyme in the liver. Increased plasma TMAO levels are associated with biological pathways that trigger endothelial dysfunction and lead to cardiovascular complications.

The biochemical pathways involved in TMAO biosynthesis, metabolism, excretion, and processes leading to endothelial dysfunction causing cardiovascular complications are summarized in Figure 1. Specifically, trimethylamine (TMA) is generated by gut microbes through dietary precursors such as choline, L-carnitine, lecithin, phosphatidylcholine, and betaine [15]. Bacterial strains involved in TMA generation include *Anaerococcus hydrogenalis*, *Clostridium asparagiforme*, *Clostridium hathewayi*, *Clostridium sporogenes*, *Edwardsiella tarda*, *Escherichia fergusonii*, *Proteus penneri*, and *Providencia rettgeri* [17]. Interestingly, individuals with cardio-metabolic diseases have an imbalance in the levels of bacteria in the gut. Elevated levels of pathogenic bacteria such as *Firmicutes* and *Proteobacteria* are found and are known to be associated with increased levels of inflammation and insulin resistance, resulting in poor metabolism [18]. In contrast, healthy individuals have a greater diversity of gut microbes that are found in stable amounts. Beneficial bacteria namely *Bifidobacterium*, *Lactobacillus*, and *Faecalibacterium prausnitzii* are present in abundant levels and are associated with improved metabolism and lower levels of inflammation [19]. Most of the TMA formed is rapidly absorbed via portal circulation [20]. In the liver, a class of hepatic flavin monooxygenase (FMO) enzymes, predominantly FMO3, causes the oxidation of TMA to TMAO [21]. Homogenous distribution of TMAO takes place throughout the body through systemic circulation, but it may accumulate in higher amounts in certain tissues [22]. In most individuals, about half of the TMAO generated is excreted without any modifications within 24 h, through urine (95%), feces (4%), as well as by sweat and breath (less than 1%) [23]. Not excreted TMAO remains circulating in the plasma, and its levels are remarkably high in patients with type II diabetes, hypertension, heart failure, and coronary heart disease [8]. In summary, these findings indicate that the gut microbiome plays an essential role in the advancement and acceleration of cardio-metabolic diseases. Therefore, understanding the species involved in TMA formation could potentially result in novel therapeutic strategies to lower the risk of these diseases. Moreover, these observations suggest that plasma TMAO levels may serve as a pre-chronic disease biomarker to assess the health status of an individual.

## 3. TMAO Detection and Measurement Methods

With the conceptual understanding that TMAO can be considered a potential biomarker for chronic diseases, detection of TMAO in plasma becomes crucial in the preliminary prognosis of several disease conditions. TMAO levels in plasma, feces, and urine samples have been analyzed [24], and commonly used methods for TMAO detection include chromatography techniques such as selective solid-phase extraction, ion chromatography, UPLC-M/MS, flow injection gas diffusion-ion chromatography, and liquid chromatography-selective ion monitoring [25,26,27,28] (Table 1). These methods are advantageous due to their analytical precision and reproducibility, but they require the expertise of specialized technicians [29], and the process is time-consuming and expensive. Other techniques involve the use of electrochemical tools such as cyclic voltammetry, differential pulse voltammetry, oxygen anti-interference membrane, and microbial electrochemical technology [30,31,32,33]. They are user-friendly and have long operational stability. However, they may be prone to environmental interferences in clinical applications. In summary, there is still a need to develop cheaper, more reliable, and more efficient testing tools to detect TMAO clinically, and identify patients with higher cardiovascular disease (CVD) risks. This will enable clinicians to intervene with the right treatment strategies and prevent the evolution of the condition.

## 4. TMAO Level Variations and Disease Conditions

Plasma TMAO levels are regulated by several factors such as age, genetics, gut microbiome, FMO3 activity, and diet [22]. For example, many studies have shown that an increase in age influences plasma TMAO levels [38,39]. Furthermore, links between various disease conditions, their progression, and plasma TMAO levels have also been established [8,40], which are summarized in Table 2. Hence, quantification and understanding of plasma TMAO levels in individuals may be essential in the pre-diagnosis of certain specific diseases. However, some findings have inherent limitations due to tight sample size, uneven gender distribution, and lack of control groups. In addition, a controversial study has shown that plasma TMAO levels may be an independent risk factor for disease conditions [41]. These different findings need to be validated by a large-scale analysis including a greater number of individuals with a balanced representation of both genders.

## 5. Molecular Mechanisms of TMAO-Induced Diseases (Table 3)

### 5.1. Endothelial Dysfunction Mediated by TMAO

**Figure 2:**
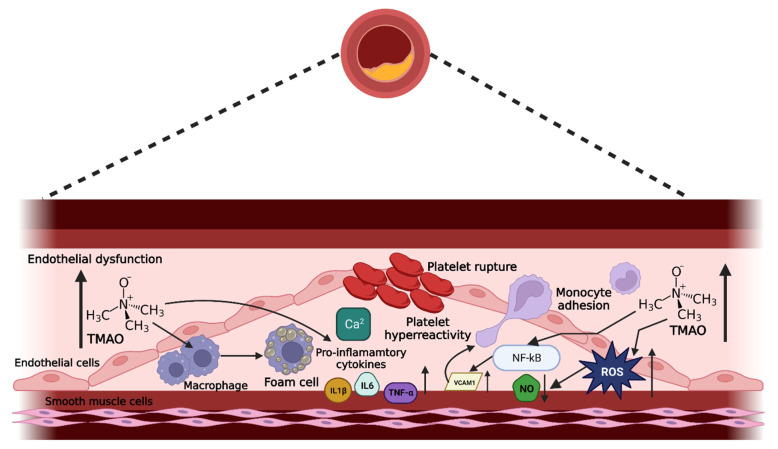
Proposed mechanisms of action in TMAO-induced cardio-metabolic diseases. Increased circulating levels of TMAO cause various processes within the endothelial cells, contributing to the pathogenesis of endothelial dysfunction and atherosclerosis.

Endothelial dysfunction, often classified as the impairment of endothelium-dependent vasodilation, is associated with oxidative stress and exaggerated activation of inflammatory pathways, which are mediated through foam cell formation, expression of inflammatory cytokines, and generation of adhesion molecules [4,6]. Endothelial dysfunction is known to play key roles in blood clotting, immune response, and vascular tone [4] (modulated via the synthesis and release of various EDRFs and EDCFs by the endothelium [4,6]), reported to contribute to CVD, CKD, and cardio-metabolic diseases such as diabetes. The proposed mechanisms of action of TMAO-activating endothelial dysfunction and triggering cardio-metabolic complications are summarized in Figure 2. These include a reduction in endothelial cell viability, overproduction of reactive oxygen species (ROS), enhanced vascular inflammation, vascular calcification leading to atherosclerotic plaques, and reduced vascular tone, which will be discussed in detail in the subsequent section. However, most studies associating TMAO and endothelial dysfunction were performed in rodents or in cell culture [53]. There is a need for clinical data to better understand the molecular mechanisms of TMAO-driven endothelial dysfunction in humans. This is crucial for the development of effective therapeutic interventions to overcome the complications of disease evolution.

#### 5.1.1. Effect of TMAO on Cell Viability

Cell viability assay is a common tool to evaluate the direct impact of TMAO exposure on endothelial cells. Despite numerous studies reporting TMAO-induced endothelial dysfunction, the effects of TMAO on endothelial cell viability remain inconsistent. For instance, TMAO (125–1000 µM) treatment for 48 h was shown to increase apoptosis in human aortic endothelial cells (HAEC) [54]. Consistent with this observation, human umbilical vein endothelial cells (HUVEC) showed lower viability after 48 h of TMAO treatment (100 µM or higher) [55]. On the other hand, several studies reported that TMAO has no significant effect on endothelial cell viability. For example, HUVEC cells treated with 10–100 µM of TMAO for 24 h did not result in any changes in cell viability [56]. Similarly, TMAO did not induce any difference in cell viability in other endothelial cell types, such as human endothelial progenitor cells [53]. This observation was consistent with another recent study where TMAO did not influence cell viability at any time point or concentration in bovine aortic endothelial cells-1 (BAEC-1) treated with 1 µM–10 mM of TMAO for 24 h–72 h [57]. Collectively, there is controversial evidence regarding the impact of TMAO on cell viability. These contradicting results may be due to the usage of different endothelial cell types, the wide range of treatment durations, and varied TMAO doses, although the range of concentrations used in these in vitro experiments were usually physiologically relevant to the plasma serum levels of patients with disease conditions (Table 2).

#### 5.1.2. TMAO Enhances Oxidative Stress

Oxidative stress is caused by the imbalance between the generation of ROS and the ability of the cells to neutralize these ROS through antioxidant activities [3,58]. Many studies have demonstrated that high TMAO concentrations induce endothelial dysfunction in cultured endothelial cells through oxidative stress [38,59]. Specifically, TMAO has been shown to trigger ROS production through thioredoxin-interacting protein- NOD-, LRR- and pyrin domain-containing protein 3 (TXNIP-NLRP3). It was demonstrated that the TXNIP-NLRP3 inflammasome complex production was activated in a time and dose-dependent manner by TMAO [60]. Another pathway responsible for oxidative stress is the Sirtuin 3 and superoxide dismutase 2 (SIRT3-SOD2) ROS signaling, which is activated by TMAO in vascular inflammation models [61]. Interestingly, TMAO lowered expression levels of SIRT1 and increased oxidative stress, both in vivo and in vitro by triggering the p53/p21/retinoblastoma tumor suppressor signaling pathways [62]. In addition, TMAO is correlated with an increase in nicotinamide adenine dinucleotide phosphate (NADPH) oxidase activity resulting in vascular oxidative stress [63]. Finally, elevated circulating TMAO levels are associated with aging in mice and humans [64], which may deteriorate endothelial cell through senescence and increase ROS generation.

#### 5.1.3. TMAO Induces Inflammation

Inflammation is a sequence of native and comprehensive immune responses that our body generates as feedbacks, upon exposure to harmful stimuli [65]. Inflammatory response, involving migration of immune cells to the damaged region, is the first step. It is followed by repair and regeneration (2nd step), involving the building of new collagen and restoration of skin homeostasis [66]. Lastly, remodeling and maturation occur to improve cellular organization where the injured tissue matures. Factors such as the overproduction of inflammatory cytokines, enhanced adhesion, and activation of foam cell formation is part of the inflammatory response [67]. Simultaneously, blood vessels present at the inflammatory site narrow down, which slows down the blood flow and activates vascular modifications [68], a phenomenon that can cause endothelial dysfunction.

##### Enhanced Cytokine Production

TMAO triggers inflammation by increasing the generation of inflammatory cytokines. Inflammatory cytokines (or pro-inflammatory cytokines) are signaling molecules generated by activated macrophages and are important players of inflammation. Some of the significant pro-inflammatory cytokines include interleukin 1 beta (IL-1β), tumor necrosis factor-alpha (TNF-α), and IL-6 [69]. TMAO is known to initiate the production of TNF-α and IL-1β [61,70], and in vitro studies confirmed the elevated levels of TNF-α production in endothelial cells, through the activation of the nuclear factor-κB (NF-κB) signaling pathway, which enhances leukocyte adhesion to endothelial walls [14]. This activates endothelial dysfunction, which may trigger CVD risks such as thrombosis and atherosclerosis [15]. In human trials, a positive relationship was also found between TMAO and IL-1β in patients with angina [53], and in a population of individuals at risk of CVD, a positive correlation was observed between TMAO levels and inflammation [71]. Collectively, data show that elevated plasma TMAO levels contribute to inflammatory and cardio-metabolic risks via the induction of inflammatory cytokines [72,73].

##### Activation of Adhesion Molecules

Relationships between TMAO and adhesion molecules have been established in the evolution of endothelial dysfunction. Expression of the vascular cell adhesion protein 1 (VCAM-1) is induced by TMAO in primary rats and human vascular smooth muscle cells (VSMCs) [74], while TMAO-induced VCAM-1 expression is triggered by the methylation of the NF-κB p65 subunit in HUVEC [56]. In fact, many studies demonstrated that TMAO-induced NF-κB activation is a significant downstream process that upregulates monocyte adhesion through upregulation of cellular adhesion molecules such as VCAM-1, but also intercellular adhesion molecule 1 (ICAM-1) and E-selectin, and enhances endothelial dysfunction [14,15]. Moreover, TMAO (10, 50 and 100 µM) is known to activate the protein kinase C (PKC) in a dose-dependent manner, which plays a crucial role in upregulating monocyte adhesion [20,75]. In summary, increased TMAO levels, in animal models and human endothelial cells, contribute to increased adhesion of monocytes and low endothelial self-repair by the activation of PKC, NF-κB, and VCAM-1 signaling pathways [56], resulting in endothelial dysfunction.

##### Elevated Foam Cell Formation

Foam cell formation is an indicative feature in the introductory phase of atherosclerosis progression, which characterizes CVD. Indeed, CVD is distinguished by inflammation-induced atherosclerotic complications, resulting from an increase in lipid particle transport to endothelial cells causing foam cell formation [76]. Foam cells (also called lipid-laden macrophages) are a key source of pro-inflammatory phenotypes as they generate inflammatory mediators such as cytokines, chemokines, and ROS, and play a significant role in activating inflammation at different stages of the atherosclerotic progression. Foam cells are formed when immune cells such as macrophages take up large amounts of cholesterol through absorption of lipoproteins via different transporters, mostly mediated by CD36, SR-A, and LOX-1. They then become overloaded with cholesterol and are unable to process it effectively. This causes these macrophages to transform into foam cells (which store esterified cholesterol and are characterized by their large and frothy appearance), which accumulate in the walls of blood vessels and contribute to atherosclerosis [77,78,79]. Studies have shown that in mice models, TMAO stimulates macrophage recruitment by promoting their migration and expression of TNF-α, IL-6 (considered promoters of foam cell formation [79]), as well ICAM1 [80]. Moreover, TMAO plays a critical role in the accumulation of ox-LDL in macrophages through upregulation of multiple scavenger receptors, CD36, lectin-like oxidized low-density lipoprotein receptor-1 (LOX-1), and class A1 scavenger receptors (SR-A1) [77], that contribute to the formation of atherosclerosis by enhancing cholesterol uptake with lipoprotein modifications [11]. This process triggers the transformation of more macrophages into foam cells within the vascular membrane [81]. Other studies demonstrated that dietary choline, a precursor of TMAO, increases foam cell production in ApoE knockout mice [11], extensively used as a model of atherosclerosis. Finally, TMAO promotes the development of foam cells by upregulating macrophage scavenger receptors [11,12,82]. Eventually, foam cell formation modulates lipoprotein metabolism and causes lesions [83]. Plaques with abundant foam cells can rupture, leading to thrombosis and CVD-related events [78]. In summary, there is a mechanistic link between TMAO and elevated foam cell generation resulting in atherosclerosis.

#### 5.1.4. TMAO Reduces Vascular Tone

Endothelial dysfunction is associated with abnormal changes in the vascular tone, which is regulated by the production of at least three vasoactive factors, nitric oxide (NO), prostaglandin I_2_ (PGI_2_), and endothelium-derived hyperpolarization (EDH) [3,58,84,85,86]. PGI_2_, one of the prostanoids of arachidonic acid metabolism, is a potent vasodilator that inhibits platelet aggregation, leukocyte adhesion, and VSMC cell proliferation [87]. NO is produced through the enzymatic conversion of L-arginine to L-citrulline by endothelial NO synthase (eNOS) [86,88]. The vasodilator actions of NO are mediated via the activation of soluble guanylate cyclase, leading to the accumulation of cGMP and the relaxation of smooth muscle cells [89,90]. Lastly, EDH is generated by contact-mediated (myoendothelial gap junctions) and non-contact-mediated mechanisms, which involve the opening of small- and intermediate calcium-activated potassium channels (SK_Ca_ and IK_Ca_) and subsequent hyperpolarization and relaxation of VSMC. Collectively, the endothelium functions normally through the production of NO, PGI_2_, and EDH to maintain vascular tone and an imbalance in these vasoactive factors result in endothelial dysfunction [91,92,93].

##### Effects of TMAO on NO Bioavailability

Studies have shown a link between elevated circulating TMAO levels and reduced eNOS, and therefore reduced NO bioavailability in the aorta of Fischer-344 rats [38]. This reduced eNOS seems to result from the upregulation of vascular oxidative stress and inflammation [94]. These data were consistent with another study where TMAO pre-treatment for 24 h significantly reduced NO production after ATP stimulation in BAEC-1, indicating the potential involvement of TMAO in damaging the endothelial-dependent vasodilatory mechanism [57]. Conversely, in the same study, TMAO pre-treatment for 1 h did not influence the intracellular NO release and eNOS phosphorylation in BAEC-1. Other findings demonstrate that eNOS activity remains unchanged in the aorta of rats treated with TMAO and in HAEC pre-incubated with 1 µM of TMAO [57]. These last findings suggest that increased plasma TMAO levels in the near-physiological range are neutral to vascular function. In summary, from these experimental results, the effects of TMAO on NO bioavailability are inconsistent, and it appears that only pharmacological concentrations of TMAO could have a negative effect in normal metabolic conditions. However, underlying metabolic diseases may interfere with TMAO effects, explaining the contradictory data from the different studies. 

##### Association between TMAO and Hydrogen Sulfide (H_2_S)

H_2_S and other vasoactive factors are key signaling molecules associated with vasorelaxation, cardio-protection, neuroprotection, and anti-inflammation. Hence, TMAO-induced NO bioavailability may potentially have a stronger effect in altering the vasculature in patients with underlying metabolic disorders, increasing their risk of endothelial dysfunction-driven diseases. H_2_S is produced in various tissues and plays a significant role in the circulatory system homeostasis, including the heart, blood vessels, and kidneys [95]. H_2_S also protects against ROS, and its proangiogenic effects can lower blood pressure and heart rate. Studies revealed that a diet enriched in choline reduces the plasma H_2_S levels, which activates cardiac dysfunction through the cyclic GMP-AMP synthase -stimulator of interferon genes-NOD-like receptor protein 3 (cGAS-STING-NLRP3) inflammasome-mediated pathway in mice [96]. However, the study did not directly measure the association between TMAO and H_2_S, hence, the direct impact of TMAO on H_2_S production and its vascular effects warrants further investigation.

##### Role of Prostanoids in Vasoconstriction

Prostanoids, metabolites of arachidonic acid, are dominant lipid mediators that modulate inflammatory responses. They include PGD_2_, PGE_2_, PGF_2_ alpha, PGI_2_, thromboxane A_2_ (TXA_2_) [88]. PGI_2_ is the most potent vasodilator prostanoid in the cardiovascular system and lowers the risk of atherosclerosis plaque formation. In mice models, choline reduces serum PGI_2_ levels and increases TXA_2_ production [97]. This causes a vasoconstrictor response and a proatherogenic phenotype, resulting in endothelial cell damage. However, there is a very limited number of studies showing the relationship between prostanoids and TMAO in causing endothelial dysfunction.

##### EDH in Endothelial Dysfunction

EDH is an essential component in small arteries, and it impacts vascular resistance, blood pressure, and the distribution flow [3]. In rats, acute treatment with TMAO specifically impairs acetylcholine-evoked EDH-mediated relaxation in the femoral arteries, indicating that TMAO contributes to the progression of peripheral arterial disease [98]. This observation is consistent with another study in rats where EDH-type relaxations were selectively disrupted without interference with NO-induced vasodilation in isolated mesenteric arteries. Taken together, these data suggest that a reduction in EDH elevates the process of endothelial dysfunction in various diseases that could be influenced by TMAO levels.

#### 5.1.5. TMAO-Enhanced Platelet Hyperreactivity

Platelet hyperreactivity is a significant factor in the activation of thrombotic environments resulting in heart attack, ischemic stroke, and severe diabetes complications [99]. High blood pressure, oxidative stress, and upregulated levels of vascular shear stress are conditions that often contribute to platelet hyperreactivity [100]. Under resting periods, platelets show a low intracellular [Ca^2+^] ([Ca^2+^]i) as they circulate through the healthy vessels [101]. However, at the site of vessel injury, platelets are activated by increased [Ca^2+^]i, a precursor to thrombus formation [102]. Physiological levels of TMAO enhance submaximal thrombin-induced augmentation of platelet [Ca^2+^]i in a dose-dependent manner [103]. In addition, the MAPK signaling pathway is a well-established driving factor of platelet aggregation by collagen [104], and TMAO causes platelet hyper-responsiveness to collagen by promoting the phosphorylation of extracellular signal-regulated kinase (ERK) 1/2 and c-Jun N-terminal kinase (JNK) [105], triggering thrombotic phenotypes [103].

### 5.2. TMAO Triggers Heart Failure

As discussed, TMAO increases the risk of atherosclerosis and CVD by different mechanisms. The terminal stage of a variety of CVD complications is heart failure (HF), a well-known cause of disability and death. The pathological mechanisms of HF are very complex, and they initiate cardiac remodeling and inflammatory responses. These processes include apoptosis and extracellular matrix accumulation, consequently causing fibrosis [106]. Animal models, such as rats and mice, have been used to study the effects of TMAO on HF. NLRP3 inflammasome activation by TMAO triggers cardiac hypertrophy and fibrosis through the suppressor of mothers against decapentaplegic 3 (Smad 3) signaling pathway [107]. In addition, TMAO triggers oxidative damage, promotes glycogen synthesis, and reduces pyruvate dehydrogenase activity as well as fatty acid β oxidation in mitochondria. This results in mitochondrial dysfunction and lower cardiac energy production [108].

### 5.3. TMAO Promotes Metabolic Syndrome

Metabolic syndrome corresponds to simultaneous disorders including hypertension, obesity, hyperglycemia, and hyperlipidemia, which increase the risk of heart disease, stroke, and type II diabetes, as well as the risk of CVD. Some of the major causes of these metabolic disorders are genetics, organ dysfunction, and mitochondrial dysfunction. A high-fat diet with TMAO precursors activates impaired glucose tolerance and inhibits the hepatic insulin signaling pathway [109]. Indeed, studies have shown that TMAO directly binds to and activates the protein kinase R-like endoplasmic reticulum kinase (PERK), causing hyperglycemia [110,111]. In addition, obesity traits are increased in mice treated with TMAO resulting in a high risk of type II diabetes, mediated by the intestinal reverse cholesterol transport and the TMA/FMO3/TMAO pathways [112]. TMAO also activates metabolic dysfunction through bile acid metabolism. It positively correlates with plasma levels of bile acids and hepatic mRNA expression of cholesterol 7 alpha-hydroxylase (CYP7A1) in mice, which trigger hepatic lipogenesis and hepatic steatosis via the bile acid-mediated hepatic farnesoid X receptor (FXR) signaling pathway [113].

**Table 3:** In vitro and in vivo models studying TMAO-linked molecular mechanisms and diseases.

Disease	Species/Cells	Molecular Mechanisms	Potential Pathways Triggered	References
Atherosclerosis and CVD	THP-1y HUVECs (Human Umbilical Vein Endothelial Cells)	↑ adhesion of monocytes, ↓ endothelial self-repair, endothelial dysfunction	Activation of PKC, NF-κB and VCAM-1 pathways	[56]
	LDLR-/- mice fed a choline diet (aorta), HAECs (Human Aortic Endothelial Cells), VSMECs (Vascular Smooth Muscle Cells)	↑ pro-inflammatory cytokines, ↑ leukocyte adhesion to endothelial wall	MAPK and NF-kB signaling pathway	[14]
	HUVECs and ApoE-/- mice (aorta)	Activation of NLRP3 inflammasome, ↑ vascular inflammation	Inhibition of SIRT3-SOD2-mitochondrial ROS signaling pathway	[61]
	Human and C57BL/6J ApoE-/- mice	↓ bile acid synthetic enzyme, ↓ reverse cholesterol transport, ↓ lipid metabolism and transport	Unknown pathway, likely to be linked to Niemann Pick Cl-like1 (Npc1L1)	[11,12,114]
	ApoE-/- mice and ApoE-/- mice with a high-fat diet	↑ macrophage scavenger receptors CD36 and SR-A1, ↑ lipid accumulation, ↑ foam cell formation	CD36-dependent MAPK/JNK pathway	[11,80,115]
	Fischer-344 rats	↑ oxidative stress, ↑ pro-inflammatory cytokines, ↑ endothelial dysfunction and vascular inflammation	Unknown	[38]
Heart failure	Male C57BL/6 mice and adult male Sprague–Dawley (SD) rats	Activation of NLRP3 inflammasome, triggers cardiac hypertrophy and fibrosis	Smad 3 signaling pathway	[116]
	Male C57BL/6 mice and cultured cardiac fibroblasts	↑ pro-inflammatory cytokines TNF and IL-1β, ↑ interstitial fibrosis in heart, ↑ myocardial inflammation, activation of NLRP3 inflammasome	NLRP3 inflammasome signaling pathway	[107]
	ICR mice and male Wistar rats	↓ cardiac energy production, ↓ pyruvate dehydrogenase activity & fatty acid β-oxidation, ↑ glycogen synthesis, ↑ oxidative damage to proteins, mitochondrial dysfunction	Cardiac energy metabolism	[108]
Kidney disease	Human and high-fat diet/low-dose streptozotocin-induced diabetes rats	↑ pro-fibrotic factors TGF-β1, IL-1β and Smad3, ↑ phosphorylation and Smad3 activation, ↑ kidney injury molecule-1, activation of NLRP3 inflammasome, renal inflammation, renal fibrosis and renal dysfunction	NLRP3 inflammasome signaling pathway, transforming growth factor β, SMAD signaling pathway	[117,118]
Metabolic dysfunction	Male C57BL/6 mice with high-fat diet (HFD)-fed	↑ insulin signaling pathway, ↑ glycogen synthesis, ↑ gluconeogenesis and glucose transport in liver, impaired glucose tolerance, ↑ insulin resistance	Hepatic insulin signaling pathway	[119]
	Human and Male ob/ob mice and wild-type C57BL/6	↑ insulin resistance, ↑ FMO3, ↑ FOX01, hyperglycemia	FMO3/TMAO pathway	[111]
	Male wild-type C57BL/6 mice	↑ hepatic FMO3 expression, TMAO activates PERK, ↑ FOX01	Stress- and PERK-related pathways	[110]
	Human and ASO-treated mice	↑ obesity traits and Type II diabetes, negative regulatory role for FMO3 in beiging of white adipose tissue	TMA/FMO3/TMAO pathway and transintestinal cholesterol excretion (TICE)/intestinal pathway of reverse cholesterol transport	[112]
	Human and cholesterol 7 alpha hydroxylase (CYP7A1) mice	↑ Cyp7a1 in mice, ↑ hepatic lipogenesis, ↑ hepatic steatosis through bile acid metabolism	Bile acid-mediated hepatic FXR signaling pathway	[113]

THP-1y: the human monocyte cell line; PKC: protein kinase C; NF-Kb: NLR family pyrin domain-containing 3; VCAM-1: vascular cell adhesion molecule-1; LDLR -/-: low-density lipoprotein receptor; MAPK: mitogen-activated protein kinases; SIRT3-SOD2: sirtuin 3-superoxide dismutase 2; CD36: cluster of differentiation 36; JNK: c-Jun N-terminal kinase; TNF: tumor necrosis factor; IL-1β: interleukin-1 beta; NLRP3: nucleotide-binding domain, leucine-rich–containing family, pyrin domain-containing-3; ICR: institute of cancer research; TGF-β1: transforming growth factor- beta 1; SMAD3: mothers against decapentaplegic homolog 3; FMO3: flavin-containing monooxygenase 3; FOX01: forkhead box protein O1; PERK: orotein kinase R-like ER kinase; TMA: trimethylamine; TMAO: trimethylamine N-oxide; FXR: farnesoid X receptor.

## 6. Potential Treatment Strategies

Understanding the involvement of TMAO in various disease conditions has resulted in active research and analyses to identify potential therapeutic strategies to reduce TMAO levels in the serum. As no specific compound directed against TMAO was found yet, a direct scavenger targeting TMAO is not available [23,120,121]. Hence, commonly proposed potential treatment strategies target the process of TMA generation, the activity of gut microbes to lower TMAO production, and the ingestion of natural products to reduce the concentration of TMAO. These therapeutic approaches are outlined in Table 4.

### 6.1. Targeting TMA and TMAO Formation Process through Enzymatic Inhibition

#### 6.1.1. Targeting TMAO

Some potential therapeutics involve the inhibition of TMAO-forming enzymes. In mice models, knockdown of FMO3 (the enzyme which converts TMA to TMAO) has been reported to suppress the expression of FoxO1 (a key protein regulating metabolism), and to prevent the occurrence and progression of metabolic dysfunction such as hyperglycemia, hyperlipidemia, and atherosclerosis. Consistent with this finding, FMO3 overexpression in mice upregulates the levels of lipids in the plasma and liver, suggesting that FMO3 may be linked to gluconeogenesis and lipogenesis, and may play a major role in glucose and lipid homeostasis regulation [138]. The drawback of FMO3 inhibition is an accumulation of TMA, which can lead to trimethylaminuria characterized by a fishy odor, and which induces inflammation. In addition, if FMO3 overexpression is closely associated with the upregulation of TMAO formation [23,82,138,139], TMAO is not the only substrate of FMO3. Hence, the inhibition of this enzyme will also lower the metabolism of other substrates such as morphine, propranolol, and tyramine [23], potentially leading to co-lateral metabolism modifications that may not be beneficial.

#### 6.1.2. Targeting TMA

Studies performed in mouse models showed that the 3,3-dimethyl-1-butanol (DMB), found in balsamic vinegar, olive oil, grape seed oil, and red wines [21], and which inhibits the choline TMA lyase enzyme [21], reduces macrophage foam cell formation and aortic root atherosclerotic lesion development in Apo E knockout mice [22,115]. In obese mice fed with a high-fat western diet, the DMB treatment does not have any effects on body weight and dyslipidemia, but significantly lowers plasma TMAO levels and prevents cardiac dysfunction. Moreover, DMB successfully prevents the expression of pro-inflammatory cytokines (IL-1β and IL-10) and TNFα. However, it is unable to completely prevent TMAO formation and does not inhibit the formation of TMA from γ-butyrobetaine [115,125]. Prevention of bacterial TMAO formation through competitive inhibition of the bacterial carnitine palmitoyl transferase-1 (CPT-1) has also been observed to be possible with meldonium. Meldonium, known for its anti-atherosclerotic and anti-ischemic properties, is an analogue of carnitine that lowers the generation of TMA from L-carnitine, but not choline, and improves endothelial function [22,127,140]. Finally, plant sterol esters can reduce the gut microbiota generation of TMA as well as cholesterol accumulation, and eliminate atherogenesis in mice [141]. However, the effects remain unclear in humans.

### 6.2. Reduction in TMAO Generation by Modifying the Gut Microbiota

#### 6.2.1. Prebiotics and Probiotics

Both prebiotics and probiotics can be used to improve the composition of the gut microbiota and regulate the level of TMAO formation [122]. Prebiotics are inclusive of all kinds of non-digestible food and are known to trigger the growth and development of useful bacteria [142], while probiotics involve the administration of living microbes that can yield beneficial effects on human health when administered in sufficient amounts, as defined by the Food and Agriculture Organization (FAO) of the United Nations [143]. Conversely, some bioactive food can reduce the generation of bacteria that convert dietary precursors in TMA. As such, the administration of *Lactobacillus paracasei* in germ-free mice colonized with human infant microbiota results in reduced TMA formation, and the use of *Lactobacillus* and *Bifidobacterium* lowers the risk of atherosclerosis [123,144]. Other studies have reported the possibility of using methanogenic bacteria (e.g., the large group of Methanobacteriales), such as *Methanomassiliicoccus luminyensis* B10, to metabolize TMA and deplete it [145,146]. In addition, probiotics lower inflammation by triggering anti-inflammatory cytokines and reducing pro-inflammatory cytokines that regulate the NF-κB pathway [147], which is linked to MAPK, pathogen recognition, and inflammatory signaling pathways [148]. The toll-like receptor expression has been shown to be downregulated by probiotics, hence also lowering intestinal inflammation [149]. However, a common limitation of probiotics used to lower TMAO levels and potentially reduce the risk of atherosclerosis is that the effect of treatment may change according to the gut microbiota composition of each specific individual.

#### 6.2.2. Antibiotics

Another strategy to lower or eliminate the conversion of dietary precursors into TMA is to target the gut microbiome composition via antibiotics. Antibiotics such as ciprofloxacin and metronidazole effectively suppress TMAO levels in clinical trials [150,151]. However, after one month of antibiotics withdrawal, TMAO levels are detected again [21,124]. Furthermore, the use of antibiotics may incur bacterial resistance or kill beneficial bacteria in addition to harmful ones [124].

### 6.3. Other Therapeutic Alternatives to Lower TMAO Concentration

Oral non-absorbent binders have been used to eliminate TMAO and its precursors. Clinically used, oral charcoal adsorbent AST-120 eliminates uremic contaminants such as indoxyl sulfate from end-stage renal disease patients [152]. However, this remains an uncertain approach as none of these absorbents specifically target TMAO [23,120,121].

Consumption of natural products may also reduce TMAO levels. Specifically, studies showed that Resveratrol (a polyphenol with antioxidant activities) modifies the composition of the gut microbiome, reducing the bacteria that promote TMA formation and increasing the useful bacteria [128,153]. *Gynostemma pentaphyllum* (an herbaceous climbing vine) lowers plasma TMAO levels and increases lecithin levels in rat models [131]. Gancao (the root of *Glycyrrhiza uralensis*) prevents the rise of TMAO levels when administered with Fuzi (the processed lateral root of *Aconitum carmichaelii*). However, it does not lower plasma TMAO levels when administered alone [60]. Oolong tea extract and citrus peel polymethoxyflavones target the TMAO formation process and lower vascular inflammation [154]. Other compounds such as berberine (BBR) [134] and trigonelline [135] are also natural products known to inhibit the formation of TMAO from TMA by lowering the expression of the FMO3 enzyme.

Anti-diabetic medications also have the potential to modify the gut microbiome. Similarly, the gut microbiota can modify the effectiveness of diabetic medications. The majority of data indicate that metformin is the most effective drug as compared to all the other anti-diabetic medications [155]. Interestingly, in db/db mice with type 2 diabetes mellitus, treatment with metformin results in a twofold reduction in TMAO concentration and the generation of bacteria associated to TMAO precursors production [137]. In this study, it was suggested that a reduction in TMAO concentration with the use of metformin is an effective therapeutic strategy to exert cardiovascular benefits.

In addition, some potential anti-obesity drugs such as capsanthin, as well as the lycopene, amaranth, and sorghum red pigments obtained from *Lycopersicon esculentum* (M.), *Amaranthus tricolor*, and *Sorghum bicolor*, respectively, also reduce serum levels of TMAO and increase microbial diversity in mouse fed with high-fat diet [129,130].

Another drug, Enalapril (ACE [angiotensin converting enzyme] inhibitor), tested in rats, increases the excretion of TMAO in the urine. However, the mechanism remains unclear, as it does not target TMAO formation or modification of the gut microbiota [21].

Despite the promising effects of these products to reduce TMAO levels, studies were only performed in mouse models. Hence, there is insufficient evidence to confirm their impact in humans.

## 7. Concluding Remarks and Future Perspective

In conclusion, the gut microbial metabolite TMAO is a significant biomarker of cardio-metabolic diseases. The molecular mechanisms underlying TMAO-induced endothelial dysfunction and subsequent development of cardio-metabolic diseases are multi-factorial, and primarily involve vascular inflammation and oxidative stress via the MAPK and NF-κB signaling pathways. Through oxidative stress and inflammation, TMAO triggers other effects such as platelet hyperreactivity and reduction in vascular tone through the impairment of EDH-mediated relaxation and PGI_2_ production. While other reported factors, such as cell viability and NO bioavailability remain controversial, the differences observed may be attributed to distinct metabolic backgrounds of models, as well as study design (cell types, TMAO concentrations, and treatment durations). Future studies should explore the molecular signatures and pathways that contribute to endothelial dysfunction and/or other cardio-metabolic diseases. While most of the current treatment strategies focus on preventing the formation of TMAO, other plausible treatment strategies could focus on targeting key mechanistic pathways that contribute to disease pathology in the various organs. Hence, a better understanding of the underlying molecular mechanisms will lead to the development of new therapeutic agents such as small molecules [156], peptides [157,158] or natural products [159,160,161] that have potent vasoprotective effects (e.g., anti-inflammatory properties) to effectively prevent or reverse TMAO-induced endothelial dysfunction and/or other cardio-metabolic diseases.

## Figures and Tables

**Table 1:** Current TMAO detection and measurement methods.

Experimental Method	Technique	Linear Range	Limit of Detection	Sensitivity	Response Time	Advantage	Limitations	References
Chromatography	FIGD-IC	40–600 nmol/dm^3^	1.35 nmol/dm^3^	-	20 min	Non-hazardous, reliable, precise (3%), sensitive	Time-consuming, expensive equipment, requires specialized technicians	[34]
GC-MS	SPME	14.9–956 μmol/L	0.01 μmol/L	14.9 μmol/L	-	Analysis of volatile and semi-volatile compounds	Complicated, laborious, time-consuming, incomplete TMAO transformation	[35]
Electrophoresis	Capillary electrophoresis with indirect UV-detection	0.025–2.5 mmol/L	2.5 mmol/L	-	-	Analytical precision, repeatability	Time-consuming, expensive equipment, specialized technicians, restrict point-of-care testing (PCOT)	[36]
Liquid chromatography	SPE	5.0–50.0 μg/mL	0.05 μg	-	-	Selective determination in presence of other primary and secondary short chain aliphatic amines	-	[25]
Chromatography	Ion chromatography	1.0–20.0 mg/mL	0.10 mg/L	-	16 min	Inexpensive and stable	Time-consuming, requires specialized technicians	[26]
Chromatography	LC-SIMs	15–944 pg/μL	115 pg/mL	-	5 min	Robust, highly sensitive, reproducible, no sample pre-treatment required, only small volume of sample needed	Expensive	[27]
Chromatography	UPLC-M/MS	15–1500 μg/L	0.12 μg/L	-	6 min	Repeatable, rapid, and economic	Not a point-of-care testing	[28]
Fluorescence	IDA	0–1.22 mmol/L	8.98 μmol/L	-	-	Low cost, easy to operate, label free, sensitive	-	[37]
Electrochemical	CV	2–110 µmol/L	2.96 nmol/L	14.16 nA/mM	16 s	Sensor can operate over prolonged daily measurements, quite good short-term usage stability	Complex preparation process (enzyme purification and protein reconstruction)	[30]
Electrochemical	DPV	1–15 ppm	1.5 ppm	2.47 µA mL/ppm/cm^2^	20 min	Easy to construct and operate, highly selective	-	[31]
Electrochemical	Oxygen anti-interference membrane	2 µM–15 mmol/L	10 µmol/L	2.75 µA/mM	33 s	Operational stability over 3 weeks	Vulnerable to environmental interferences in clinical applications	[32]
Electrochemical	Microbial electrochemical technology	0–250 µmol/L	5.96 µmol/L	23.92 µA/mM	600 s	90% accuracy in real serum, high feasibility in clinical applications	-	[33]

FIGD-IC: flow injection-ion chromatography; GC-MS: gas chromatography-mass spectrometry; SPME: solid-phase microextraction; SPE: solid-phase extraction; LC-SIMs: liquid chromatography-selective ion monitoring; UPLC-M/MS: ultraperformance liquid chromatography tandem mass spectrometry; IDA: indicator displacement assay; CV: cyclic voltammetry; DPV: differential pulse voltammetry.

**Table 2:** Association of plasma TMAO levels with various disease conditions.

Experimental Model	Condition (If Any)	Plasma TMAO Level for Control	Plasma TMAO Level for Condition	References
Human and Rodents	-	0.5–5 µmol/L	-	[11,42]
Human	Patients undergoing hemodialysis	0.92–1.9 µmol/L	28–67 µmol/L	[43]
	Chronic Kidney Disease	-	32.2–75.2 µmol/L	[44]
	Inflammatory Bowel Disease	-	2.27 µmol/L	[45]
	Plaque rupture	4.2 ± 2.4 μmol/L	8.6 ± 4.8 µmol/L	[46]
	Calcified aortic valve disease	1.4–2.8 µmol/L	2.3–6.4 µmol/L	[47]
	Stage 1 hypertensive patients	-	87.2 ng/mL	[48]
	Older age, BMI, lower eGFR, HDL-levels, higher choline and carnitine levels, higher TG	2.83 ± 1.34 μmol/L	8.43 ± 4.85 µmol/L	[49]
	Stroke	1.4–3.7 µmol/L	1.6–4.0 µmol/L	[50]
	First ever acute ischemic stroke and neurological deficit	2.6–6.1 µmol/L	0.5–18.3 µmol/L	[51]
	Stroke (LAA), transient ischemic attack, history of diabetes, CAD, HBP, HLP	1.91 µmol/L	2.70 µmol/L	[52]

BMI: body mass index; eGFR: estimated glomerular filtration rate; HDL: high-density lipoprotein; TG: triglycerides; LAA: large-artery atherosclerosis; CAD: coronary artery disease; HBP: high blood pressure; HLP: hyperlipidemia.

**Table 4:** Proposed therapeutic approaches to target TMAO formation.

Intervention	Therapy	Model	Intervention/Dosage	Duration	Route of Administration	Effects	Limitations	Reference
Targeting the gut microbiome	Prebiotics	Human	Whole grains, traditional Chinese medicinal foods, and prebiotics (WTP diet)	30–90 days	Oral	Improves composition of gut microbiota to reduce TMAO formation	Gut microbiome is influenced by multiple components	[122]
	Probiotics	Female germ-free mouse (C3H strain)	Basal mixed diet and probiotics supplementation in saline water	14 days	Oral	Lowers TMAO formation in the gut	Unclear safety and effects in humans	[123]
	Antibiotics	Mouse	Drinking water with a cocktail of broad-spectrum antibiotics	21 days	Oral	Suppression and inhibition of plasma TMAO levels	Inhibition of useful bacteria and induction of resistant bacteria. Not feasible in the long run	[11]
	Antibiotics	Human	Metronidazole (500 mg twice daily) plus ciprofloxacin (500 mg once daily) for 1 week	7 days	Oral	Suppression and inhibition of plasma TMAO levels	Inhibition of useful bacteria and induction of resistant bacteria. Not feasible in the long run	[124]
	Oral non-absorbent binders	Human and rat	10 mL solution of 800 mg of polymyxin B and 320 mg of tobramycin (selective decontamination of the digestive tract [SDD])	56 days	Oral	Removal of TMAO and its precursors from gut	Uncertain approach. Compound specific to TMAO has not yet been found	[121]
Targeting TMA formation	Inhibition of FMO3	Wild-type C57BL/6J, male Sprague–Dawley rat (Harlan) and human	50 mg/kg body weight of antisense oligonucleotides (ASO)	7 weeks or 16 weeks	Intraperitoneal injection	Inhibition of TMAO formation from TMA	Accumulation of TMA in plasma may cause other diseases. Metabolism of other compounds is also mediated by FMO3	[111]
	3,3-Dimethyldimethyl-1-butanol (DMB)	Mouse	1% DMB in drinking water	56 days	Oral	Inhibition of TMA formation from dietary precursors choline, carnitine, corotonobetaine by inhibiting microbial TMA lyase	Complete TMAO formation cannot be avoided by DMB. Study not performed in humans. Unable to inhibit formation of TMA from γ-butyrobetaine	[125]
	3,3-Dimethyldimethyl-1-butanol (DMB)	Mouse	1%, *v*/*v* in drinking water	16 weeks	Oral	Reorganization of gut microbial community and inhibition of TMA production	Only partial TMAO formation inhibition	[115]
	Meldonium	Human and rat	Single dose of ^13^C-GBB (100 mg/kg) or ^13^C-GBB in combination with meldonium (GBB + M, 100 mg/kg each)	14 days	Oral	Lowers TMAO formation from L-carnitine and increases TMAO excretion via urine	Unable to reduce TMAO formation from choline	[126,127]
Therapeutic alternatives to lower TMAO concentration	Resveratrol	Female C57BL/6J mouse and ApoE-/- mouse with a C57BL/6 genetic background	0.4% RSV	30 days	Oral	Alters gut microbiome composition, hence reducing bacteria that forms TMA and increasing useful bacteria	Study performed only in mice. No changes when antibiotics are used	[128]
	Capsanthin	High-fat-diet induced obese C57BL/6J mice	Capsanthin at 200 mg kg^−1^ body weight	12 weeks	Oral	Lowers body weight, effectively reduces TMAO levels, and increases microbial diversity	Study performed in mice	[129]
	Lycopene (*Lycopersicon esculentum* [M.]), amaranth (*Amaranthus tricolor*), and sorghum red (*Sorghum bicolor* (*L.*)) pigments	High-fat diet fed C57BL/6 mice	200 mg/kg body of lycopene or amaranth or sorghum red administration	12 weeks	Oral	Ameliorates lipid metabolism, and lowers TMAO levels	Study performed in mice	[130]
	*Gynostemma pentaphyllum*	Rat	120 mg/kg/day	28 days	Oral gavage	Lowers plasma TMAO levels and rises in lecithin levels	Study performed only in rats	[131]
	Gancao (root of *glycyrrhiza uralensis*)	Male Wistar rat	Single dose of Gancao (35.6 g kg−1 body weight)	-	Intragastric administration	Prevents increase in TMAO when administered with Fuzi (processed lateral root of *Aconitum carmichaelii*)	Does not lower TMAO levels when administered alone. Study performed in rats	[132]
	Oolong tea extract and citrus peel polymethoxyflavones	Mouse	1 μg	Injection every 10 days for a period of 16 weeks	Intravenous injection	Lowers TMAO formation and vascular inflammation	Study performed only in mice	[133]
	Berberine (BBR)	ApoE-/- mouse on a C57BL/6 background	BBR treatment (50 mg/kg) twice weekly	84 days	Intragastric administration	Lowers expression of hepatic FMO3 and serum TMAO levels	Study performed only in mice	[134]
	Trigonelline	C57BL/6 J mouse	Trigonelline (50 to 100 mg/kg) per day	14 days	Oral	Inhibits conversion of TMA to TMAO by inhibiting FMO3	Study performed only in mice	[135]
	Enalapril	Rat	5.3 or 12.6 mg/kg	14 days	Oral	Increases TMAO excretion in urine	Unclear mechanism. Does not affect TMA formation or composition of gut microbiota	[21,136]
	Metformin	db/db mice	250 mg/kg/day	8 weeks	Oral	2-fold reduction in TMAO levels and bacteria linked to TMAO production	Study performed in only mice	[137]

## Data Availability

Data sharing not applicable.
